# Social learning as an underlying mechanism for sustainability in neglected communities: The Brazilian case of the Bucket Revolution project

**DOI:** 10.1007/s10668-022-02167-z

**Published:** 2022-02-26

**Authors:** Michelle Bonatti, Carla Erismann, Ayna Askhabalieva, Juliano Borba, Kamila Pope, Renata Reynaldo, Luca Eufemia, Ana Paula Turetta, Stefan Sieber

**Affiliations:** 1 Leibniz Centre for Agricultural Landscape Research (ZALF E. V), 15374 Müncheberg, Germany; 2 Department of Agricultural Economics, Humboldt University of Berlin, 10099 Berlin, Germany; 3 Institute of International Relations and Defense (IRID/UFRJ), Federal University of Rio de, Janeiro, 250 Pasteur Avenue, Campus Praia Vermelha, Rio de Janeiro, Brazil; 4 Brazilian Agricultural Research Corporation (EMBRAPA Soils), Rio de Janeiro, 22460-000 Brazil; 5 Program of Territorial Development and Public Policy, Federal Rural University of Rio de, Janeiro, Rio de Janeiro, Seropedica 23890-000 Brazil

**Keywords:** Socio-ecological innovation, Transformative learning, Community-based food systems, Triple-loop learning, Endogenous social learning

## Abstract

In neglected communities, waste and organic residues are not only a vector of several problems, like diseases and water pollution, but also a contributor to increasing forms of vulnerability and marginalization. At the same time, these communities also have presented innovative local initiatives and transformative learning about natural resources management that can be a vehicle for achieving more sustainable food systems. In the south of Brazil, community-based organic residue management has shown an extraordinary potential to improve food security and livelihoods for (≈1600) community members of a vulnerable urban territory. In this context, the overall objective of this article is (a) To better understand what Social Learning (SL) processes related to successful organic residues management in neglected communities exist and (b) To identify what knowledge systems are created in one empirical case. The study case is based on a communitarian waste management project, the Bucket Revolution Project (BRP). The analytical framework builds upon social learning theory and its triple-loop process focusing on four specific phenomena. The applied mixed-methods approach was made in four steps: 1. a focus group to investigate collective community issues; 2. semi-structured interviews to investigate specific and individual issues in the context of the BRP; 3. social media analysis to better understand the BRP narratives; and finally 4. participant observation in community and institutional meetings. Mainly using MaxQda software and coding indicators of SL, the data show that “Diversity of knowledge integration” is the most identified SL indicator in the interviews (52%). For BRP, identity development, community conditions improvement, and environment understanding are three key components of the knowledge system enhanced through an underlying process of social learning. Furthermore, the study also shows that there are endogenous and exogenous social learning processes at work.

## Introduction

Community-based organic residues management has proven potential to effectively recycle, improve residues flow, and promote new kinds of sustainable food systems (Abreu, 2013; Pope, 2020). It promotes fork-to-fork cycles that interconnect local composting and food production in urban and rural areas (Turetta et al., 2021). Although community composting is common in some countries, there is still a lack of information on the enabling conditions necessary to optimize logistical, environmental, economic, and social impacts (Bruni et al., 2020), especially in vulnerable communities. The existent practical cases are neither explored nor systematized in the literature, most likely because many of the “real-world” experiences are led by community members and rarely entirely led by researchers. In this context, understanding how innovative community-based organic residue management is developed among community members is crucial for “transferability” and dissemination.

In neglected communities, waste and organic residues are not only a vector of several problems, like diseases and water pollution (Ayilara, 2020), they are also a contributor to increasing (forms of) vulnerability and marginalization. Neglected communities are populations that systematically do not receive enough services from the government. These territories face a lack of several human rights, living an overlooked poverty with insufficient clean water and sanitation, health services, and education, just about every facet of development (Jacobson and Bush 2018). As it occurs in poverty traps, these territories experiment reinforcing cycles of vulnerability, where lack of human rights (i.e. education, waste treatment/public sanitation) leads to different aspects of vulnerability; as low-income conditions leads to poor household infrastructures, which leads to problematic environmental conditions, diseases and, in many cases, also results in social discrimination.

As a reaction to these vulnerable reinforcing cycles, these communities also have presented significant local initiatives and transformative learning that can be a vehicle for achieving more sustainable waste management and food systems. In Brazil, experiences of organic residue management in vulnerable and food-insecure communities are bringing empirical evidence showing options for the better design of sustainable waste management (Tremblay & Gutberlet, 2012) and urban agriculture (Abreu, 2013). While the country faces a historical process of negligence in tackling environmental problems related to waste management, it also presents transformative community-based models (Tremblay & Gutberlet, 2012). For instance, the urban agriculture initiative and Latin America’s largest community farm, *Horta de Manguinhos* project (Manguinhos vegetable garden), created in 2013, is helping at least 800 families survive the coronavirus outbreak, as well as employing more than 20 local workers. As pointed out by Rekow (2017), this initiative demonstrates how vulnerable contexts can benefit from creating green infrastructure that is anchored by community involvement and collective action. In southern Brazil, *A Revolucao dos Baldinhos* (the Bucket Revolution Project (BRP), created in 2009, collects domestic organic waste for use in urban agriculture in vulnerable areas of Florianópolis, Brazil. In its first four years, this waste management system treated 1200 tons of organic waste and contributed to the production of nutritious food of participating families, benefitting over 1600 people (Abreu, 2013).

These are strong examples of effective organic residue management implemented through community transformative power. They can show how to address sustainability challenges, how complex problems can be tackled, how they can be better framed in neglected communities’ reality, and how to engage community members in learning systems development. In this context, the overall objective of this article is to better understand what social learning processes related to successful waste management in neglected communities exist and to identify the knowledge systems created in one empirical case. The study case is the Bucket Revolution Project (BRP). The project is based on a cooperation model focusing on the recycling of biomass and promoting urban agriculture (UA) in the peripheral region of Florianópolis, Brazil.

The analytical framework to better understand the collective knowledge systems of this initiative is centred on social learning theory (Wals, 2007; Collins & Ison, 2009). Social learning is based in collaborative groups and networks that: (a) Integrate different sources of knowledge; (b) Undertake iterative and transformative planning and management change in response to new learning and information; and (c) Ensure that there is an impact from such collaborative efforts. Further, social learning should demonstrate the emergence of an understanding that goes beyond individuals or small groups, including both wider social units (Reed et al., 2010) and communities of practice (Wegner, 2000, Wegner, 2011).

Although definitions vary (Ison et al., 2013), in a nutshell, social learning approaches help facilitate not just knowledge sharing and knowledge co-creation between diverse stakeholders around a shared purpose, but it also takes learning and behavioural change beyond the individual to networks and systems (Kristjanson et al., 2014). Social learning is largely associated with improving sustainability problems (Medema et al., 2014, Wals, 2007; Bonatti, 2018). It is frequently proposed as a fundamental shift in the ways people work by using more humane tools when advancing individual and collective achievement (Bingham & Conner, 2010) as well as engagement processes that cope with the complexity and uncertainty facing natural resources managers (Holling, 1978).

Sustainable development projects in neglected communities tend to be more effective when they are based on self-organizing learning trends, critical reflection, and processes that aim to encourage people to act together (De los Rios et al., 2016; Reed et al., 2010; Bonatti, 2018). In this context, this article focuses on two research questions: (1) How is SL practically developed and based on which knowledge systems? And (2) To what extent do social learning processes occur in the BRP, specifically, which kinds of social learning levels/orders occurred? The hypothesis is that the social learning process was essential to organize the knowledge systems related to environmental issues at the community level and beyond.

To analyse the occurrence of social learning in the BRP case, this research focuses on the occurrence of the following phenomenon:

(A) A change in understanding occurs, and individuals are involved: from simple processes like recalling information to higher levels like changes in worldviews, beliefs, and attitudes.
(B) A learning process that moves beyond the individual level to a broader social context (communities of practice), occurring via interactions within a social network.
(C) The convergence of goals (more usefully expressed as agreement about purpose), criteria, and knowledge leading to awareness of mutual expectations and the building of relational capitals.
(D) Occurrence of a learning process with triple loops: a. Hindsight—Information reception and application; b. Insight—Reflecting on effectiveness and efficiency; and c. Foresight—Resulting in transformational change in other situations (Harvey et al., 2012; Tosey et al., 2012).

King and Jiggins (2002) explain that single-loop learning generates knowledge from doing, whereas double-loop learning explores the underlying values and assumptions behind our knowledge and learning. Triple-loop or multi-loop social learning is recognized as a crucial element for natural resources management involving a process of managing change where the central methodological concern is with effectively engaging the necessary participation of system members in contributing to the collective knowledge of the system (Medema et al., 2014).

## Methodology

### Description of the case of study

**Fig. 1:**
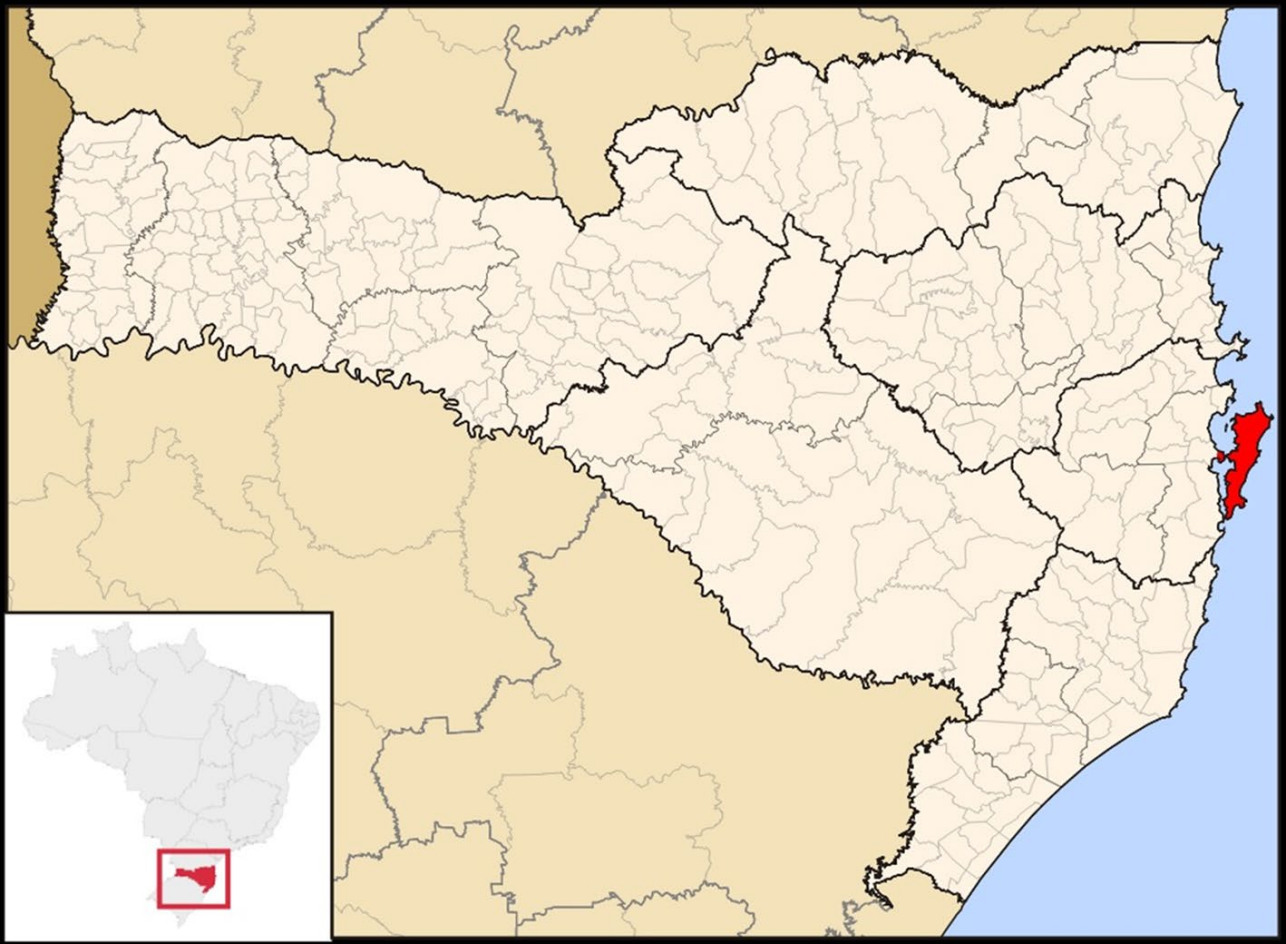
The city of Florianopolis, Brazil. Source: Adapted from Wikipedia.com

The city of Florianópolis, Brazil (Fig. 1), where the case of study is located, has been growing at a very fast pace since the 1990s, and the city government has struggled to settle population and provide basic services. Nevertheless, state programmes emphasize services to the wealthy areas and not to the peripheries (Abreu, 2013). Thus, the poorer communities suffer more drastically from the city’s unplanned growth than wealthier areas.

The peripheral community “Comunidade Chico Mendes”, where Bucket Revolution Project was started, is in the continental region of Florianópolis and is one of the most vulnerable communities of the city. The population of the community mainly comprises people who migrated from the countryside or other regions of the country to the city in order to improve their livelihoods. The community started in 1970 with the occupation and appropriation of the area. Since then, many different urbanization projects have been carried out in the community, but some problems persisted, such as the poor management of organic waste, which causes bad smells and infestations of rats, cockroaches, mosquitos, and other disease vectors. Rats were especially present in this area and, in 2008, two people died from Leptospirosis (disease spread by rats and other rodents) in the community of Chico Mendes (Abreu, 2013).

After these two deaths and the initiative of local actors to discuss the risk of disease vectors, the population gained increasing awareness of the problem of uncollected and badly managed organic waste in the area. In January 2009, with the collaboration of an NGO and the Federal University that were already organizing and implementing urban agriculture in regional schools, the project was started to be elaborated; by July—6 months later—there were already 95 families taking part in the project (Gama, 2019). Although the number varies due to the lack of public data collection, as of spring 2021, the project engages 150 families directly.

The composting process has generated several benefits to the community. Besides of improving health conditions, by turning food “waste” into compost, the organic residues are transformed into precious nutrients back to the soil. Composting is a biological process that occurs under aerobic conditions (presence of oxygen), and requires adequate moisture and temperature in order to transform organic wastes into a homogeneous and plant available input (Román et al., 2015). Compost is used to improve soil structure through the addition of carbon and provide plant nutrients. In addition to being a source of plant nutrients such as nitrogen (N), phosphorus (P) and potassium (K), it improves the physicochemical and biological properties of the soil. In the community, compost is essential to the home and school gardens. In this sense, compost can compensate for the lack of fertilizers and improve food production.

From a socio-environmental problem and the desire to decrease and prevent the incidence of rats in the vicinity, the “Bucket Revolution Project” (BRP) emerged as a movement for the separation and collection of organic waste from households in buckets and recycling through thermophilic composting at the local public school. The basic mechanism of the project is based on the distribution of buckets to the families living in the community. They fill the buckets with household organic waste and then bring it to the “ponto de entrega voluntária” (site for collecting the organic waste, where all the biomass arrives at first). Moreover, twice a week, the collected organic waste is brought to the composting site. The collaborators carrying out the composting are mainly voluntary local school students and the local agents. Once ready, the compost is distributed to local gardens and sold to other communities.

Although several participants are students, the relationship between BRP and the local public school *América Dutra Machado* was unstable. This included the relationship between teachers and students that oscillated in quality as well as in the level of cooperation in different stages of the project. The school hosted the space for a composting and urban agriculture project coordinated by CEPAGRO when, in 2008, the leptospirosis epidemic emerged, which led to the emergence of BRP. And all the while, the school dedicated part of its space to recycling organic waste. However, acceptance among teachers was not unanimous.

### Methods

**Fig. 2:**
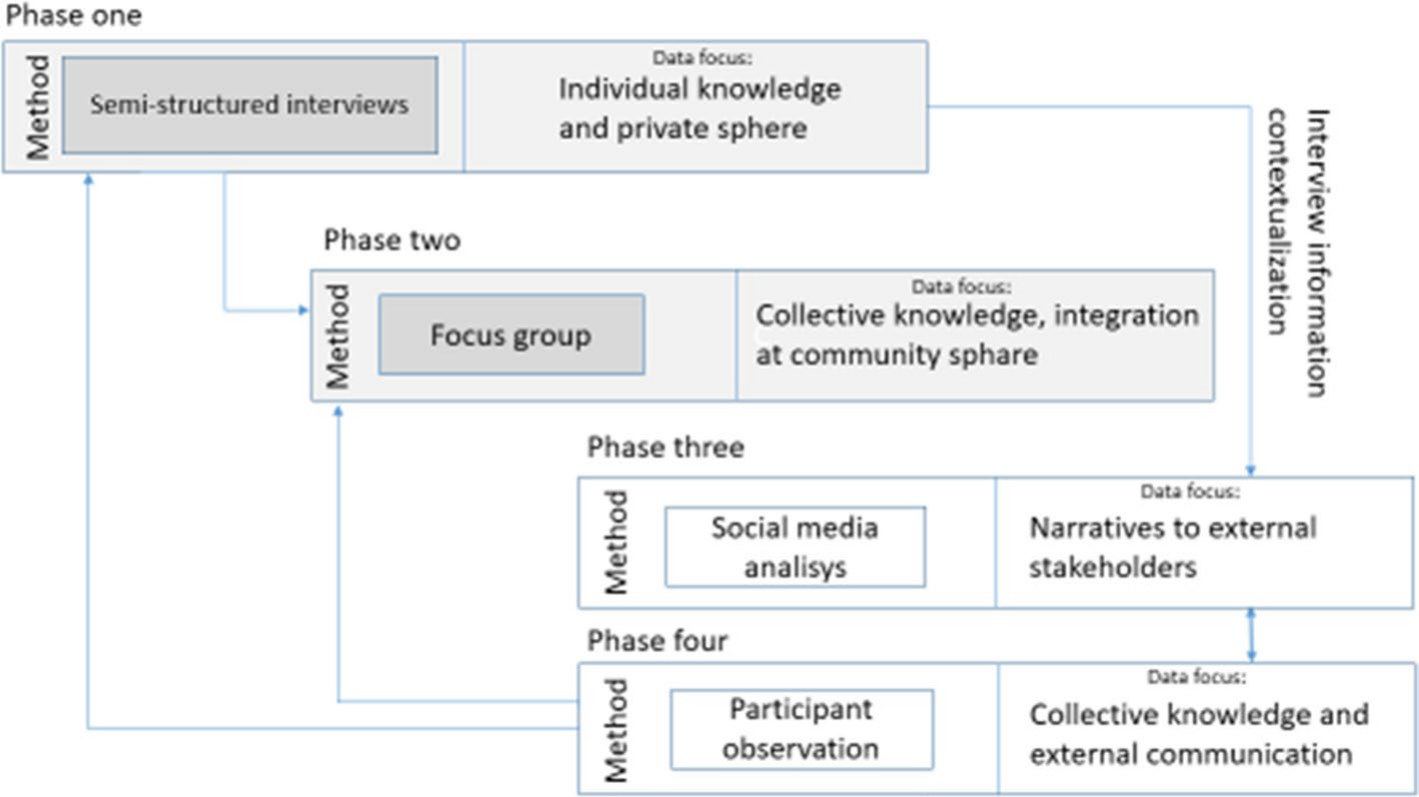
Methodological framework comprising four phases to analyse the different levels of knowledge and learning

The methodological framework (Fig. 2) is based on community-based research and critical communicative methodology (CCM). As pointed by Gómez (et al. 2011), CCM is a methodological response to the dialogic turn of societies and sciences to transforming situations of inequality and exclusion. Research conducted with the CCM implies continuous and egalitarian dialogue among researchers and the people involved in the communities and realities being studied. This research model involves, first, a dialogic collaboration (Lentz, 2018) with BRP members in order to identify key factors to explore in this research. This methodological approach was adopted to better understand the community context and the constraints that BRP members themselves identified as essential and then, subsequently, dive in-depth into the mechanisms of social learning. Furthermore, community members were involved in the data collection. The involvement of community members was also crucial to avoid disconnections in knowledge development and potential conflict. Therefore, by community members’ engagement, different views constructed the knowledge presented in the research. The dialogic approach also gave community members space to report their frustration about not being adequately recognized by academia in other previous experiences (Fig. 2).

The applied mixed-methods approach comprised four phases: 1. Semi-structured interviews to investigate specific and individual issues in the context of the BRP; 2. focus group to investigate collective community issues; 3. social media analysis to better understand the BRP narratives; and 4. participant observation in community and institutional meetings. The core of the data collection comprised phases 1 and 2. Therefore, phases 3 and 4 served to better contextualize the result from phases 1 and 2 (Fig. 2).

The focus group (1) was conducted as a collective dialogue following semi-structured questions (open end). The questions focused on understanding the problems faced by the community (significant community issues), the trajectory of the BRP, the social actors who are involved, and how they were involved, as well as what was learned. The participants (*n* 10) were divided into four categories: current active member of BRP; past active member of BRP; long-term passive member of BRP; and recent passive member of BRP. Active members are those working in decision-making and organic residue management (collecting and composting). Passive members are considered the families who give their organic residues to be composted. The focus group lasted 150 min. The discussions were recorded and transcribed with the participants’ consent. The analysis of data was done in the original language, Portuguese.

For phase (2), a total of 15 semi-structured interviews were conducted. The interview guide comprised 35 questions that were adapted accordingly to the interview. The questions were divided into five subsections: identification, community problems, learning process, governance, and community transformation. The idea of having these categories was to avoid asking direct questions about how much and in each topic people have learned but rather to capture if people mention it naturally. By taking this approach, it ensured that participants were not induced into providing the desired or expected results that would prove that social learning occurred.

To represent the diversity of actors, two samples were drawn. The first comprised those who were either a past or current member of the BRP; the second included representatives of institutions directly influencing waste management in Florianopolis city. Sample 2 comprised individuals from research/academic institutions, policymakers, and professionals from the public waste management institutions in Florianopolis. The first sample included 80% of active members directly leading the project. This first sample was compounded by women, low-income, and aged between 20–60 years old.

Each interview lasted about 60 min. They were recorded and transcripted with the participant’s consent. The collected data in the focus group and interviews was analysed with MAXQDA software, a program designed to analyse qualitative data, thus belonging to the CAQDAS family, or “Computer Assisted Qualitative Data Analysis Software”. Using MAXQDA analysis, codes—a group of meanings—were identified.

**Fig. 3:**
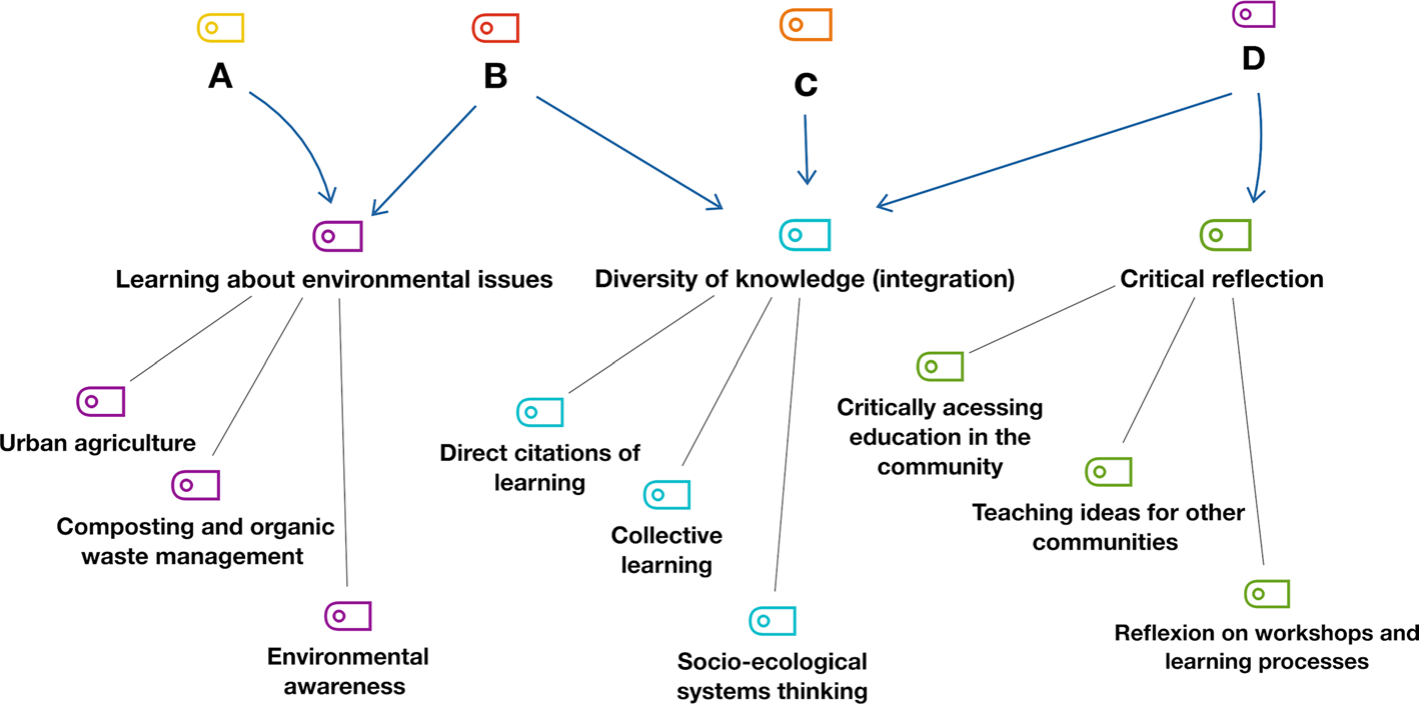
Code system for the analysis of the interviews and focus group data. Subtitles: A—A change in understanding occurs, and individuals are involved; B—The convergence of goals; C—Learning process that moves beyond the individual level; D—Occurrence of a learning process associated with triple loops

The code system to analyse the data collected was based on the four phenomena of social learning presented in the introduction. Logical three (Fig. 3) was created to represent the schemes of knowledge areas and their interconnections, providing indicators for the research questions (Fig. 3).

The systems of codes were divided into three main groups and nine respective subgroups (three each) (Table 1).

**Table 1:** Systems of codes

Codes based on the 4 SL phenomena	Subcodes
1—Learning about environmental issues	Urban agriculture Composting and waste management Environmental awareness
2—Diversity of knowledge (integration)	Direct citations of learning (I learn…) Collective learning Socio-ecological systems thinking
3—Critical reflection development	Critical education in/about the community Teaching ideas for the other communities-Reflection on the educational activities (external workshops) and learning process

For methodological phase 3, social media analysis was employed to better understand the BRP narratives. Social media (SM) analysis is a relevant method because it holds the potential for creating a virtual environment where social learning can take place by watching other users’ attitudes, behaviours, and values (Lorenzo et al., 2012). Thus, SM users can learn through the environmental and ecological experience and knowledge of others and in this way, provoke attitudes towards environmental responsibility (Zafar et al, 2021). The SM complementary method was based on discourse analysis of the available online content of the BRP. The selected SM was Instagram, giving its high level of interaction between users. Discourse analysis was selected as a method because it provides a systematic way to analyse communication data and integrate the different media types, including photos, videos, and written content (Müller & Geise, 2015).

Although DA was originally used in the field of cognitive psychology (especially Potter & Wetherell, 1987), now it is applied to multiple fields (Wooffitt, 2005). Even though DA typically focuses on the linguistic terms and micro-details of interaction, in this study, we use a broader view and do not make an in-depth analysis of the linguistic peculiarities of the discourses. Instead, we use some references of linguistics repertoires in the posts and analyse it as in accordance with a rather sociological perspective introduced by Foucault (Wooffitt, 2005).

To gather data from Instagram, first, the 70 most recent posts tagged as “learning” were evaluated. This analysis was designed to understand networking (directly related to practice, community, and learning) as well as common topics that are presented as linked to the BRP. The topics and the content as the characteristics of the users tagging the BRP Instagram were analysed. Here, in a first analysis, tagged posts by the BRP profile are not considered since it is not a sign of networking. This offers a more “universal” view of how BRP public relations take place on social media and what are the most common topics. The analysis was done by creating an Excel table with all written content copied alongside short descriptions of posted photos and videos.

The second analysis examined BRP posts on Instagram. By analysing their direct posts, it is possible to capture their discourse and the main understanding of how they might affect the construction of senses from not just their online followers, but also their direct community since many of the BRP posts are directed to the community’s population. In this sense, this analysis allows a more internal impression of BRP narratives, whereas the analysis of tagged photos offers a rather external analysis. To create a systematization of the data gathered, the following protocol was created. As of 16 July 2020, there were 312 posts. The browser visualization of Instagram offers rows of three photos, meaning 110 rows. To reduce it to a number that is possible to analyse, the first photo of every second row was selected, giving a final sample of 55 posts. The decision is based on the premise that it would be essential to have historical data, mainly due to COVID-19. The pandemic caused an overflow of posts regarding health measures, best practices, and community organization. This data is also taken into account. Nevertheless, COVID-19 is an unusual situation that causes many media to shift from their original topics to health concerns, meaning that historical data offers a better range of analysis.

Methodological phase 4 consists of participant observation in community and institutional meetings at eight meetings. Participant observation is a primary ethnographic research strategy designed to give the researcher a close and intimate familiarity with a given area of study (such as religious, occupational, or social rules) through involvement with people in their natural environment and not characterizing one–one communication. Here, participation typically involves a range of methods, including informal and unstructured interviews, direct observation, and participation in the life of the group or collective discussions/dialogue. Results of this methodological phase were used to support or contrast the results of previous phases (Fig. 2). The use of different qualitative methods is intended to create a complementary source of data to assess social learning processes from different perspectives. The social media analysis allows a rather public and content-focused analysis, as well as online interactions. Given technological advances and the pandemic, social interactions and social learning evidence may potentially be observed via online platforms.

## Results

Results are presented by exploring the frequency (%) of codes and subcodes identified in the interviews and focus group through the MAXQDA software as well as statements by the participants that reinforced these findings.

### General results

As an overview, results show that knowledge about organic residues management was developed and advanced collectively due to BRP activities. The interviewees show a clear understanding of waste management, the ecological process involved, and other sustainability issues. Furthermore, this knowledge is directly connected with a critical analysis of general community conditions and human rights.

Tables 2 and 3 summarize the frequency of the codes identified in interviews and focus group. According to the analysis of the data collected in these methodological phases, code group 2, “Diversity of knowledge (integration)” is the most identified in the interviews (52%) (Table 2). The strongest relevance of this result is that the code group has the most connections to the social learning phenomena (A, B, C, and D), based on the arrows in Fig. 2. Code groups 1 and 3 (learning about environmental issues and critical reflection), represented 25.25%, and 23.20%, respectively.

**Table 2:** Results for the code group’s frequency and percentage

Coding groups derived	Frequency	Percentage %
1—Learning about environmental issues	65	25.25
2—Diversity of knowledge (integration)	133	51.55
3—Critical reflection	60	23.20
Total	258	100

**Table 3:** Summary of results for codes and subcodes frequency/percentage

Frequency of the Coding groups	Subcodes analysis
1—Learning about environmental issues (25.25%)	Urban agriculture (49.2) Composting and waste management (26.2) Environmental awareness (24.6)
2—Diversity of knowledge (integration) (51.55%)	Direct citations of learning (I learn…) (22.6) Collective learning (62.4) Socio-ecological systems thinking (15)
3—Critical reflection development (23.20%)	Critical education in/about the community (38.3) Teaching ideas for the other communities (Least identified) (10) Reflection on the educational activities (external workshops) and learning process (51.7)

Results from the categories of interviewees “past or current member of the BRP” show a high percentage of learning schemes.

### Results according to specific codes and subcodes

#### Code 1

“Learning about environmental issues” is the second most frequent subcode. It demonstrates an overall understanding of environmental issues, even when related to other aspects, as exemplified by the words of Interviewee 12, a BRP member:

*The heart of the BRP is the cycling of food and organic waste management, the learning processes go beyond the core topic. Moreover, it is important to remember that the primary motivation of the BRP originates from a health security issue in the community, a basic need they faced. Thus, the trend of evaluating and acquiring knowledge by the community remains based on their necessities, and but not all on an environmental agenda*.

In code 1, the most frequent subcode was “urban agriculture” (49.2%) (Table 2). The interviewees, especially BRP members, mentioned urban agriculture as a path of resistance and of economic empowerment. Moreover, the topic is linked to the fight for territory in the city, at an ideological level. One interviewee reports how one of the gardens was threatened and how she had to fight to keep it but lost the fences they had constructed. Another interviewee reports not having enough space or sunlight at home to even be able to have a vertical garden at home, but still was looking forward to practicing more urban agriculture.

*They can better use this tool that is the Bucket Revolution. Through urban agriculture, composting we open a range of possibilities and have this strengthening of the territory, but from there goes beyond the methodology (Interviewee #12).*

*Urban agriculture is a good alternative for people to have at least subsistence... In a situation of so much unemployment that one has at least the subsistence… we have to fight (Interviewee #2).*

The subcode “Composting and organic waste management” has a frequency of 26%. Recurrent statements about the use and importance of compost for planting in home gardens were identified. Moreover, the participants highlighted economic gains since they do not buy fertilizer, buy less food, and learned how to produce themselves.

*Our community has changed a lot. About street cleaning, there's no more rubbish on the streets, right? And another, the fertilizer, right? Fertilizer, right? Fertilizer helps a lot (Interviewee #3)*

*(we) have waste but you already know to turn into a dish, to turn into a tomato sauce, to turn into candy, and that mother brings to income generation. So, we have created a range in our work with the health centre, which has had a workshop, a visit to the health ministry, and sometimes made health syrups, making mint drops. These are other things. The question of food and composting also inspire actions in this sense. And BRP has been working on more… (Interviewee #6)*

The results linked to the subcode “environmental awareness” of the interviewees are linked with a holistic view of organic waste management and its social implications. Moreover, the changing behaviour of the community is often cited by the interviewees. These changes include political understanding and speech, as well as engagement in urban agriculture, changing behaviour towards waste management in general, engaging socially and politically in the community activities.

*Our community has changed a lot. About street cleaning, there's no more rubbish on the streets, right? And another, the fertilizer, right? Fertilizer helps a lot. Like, I plant, I have a lot of lettuce, marjoram, onions, a lot of things I've planted, you know. And it's a very good thing. And then the soap here, it's a very good, very good thing (Interviewee #1).*

*…there is a clear understanding of the cycle of organic waste as the creation and transformation into food all the movement that Cintia (community leader) and the revolution influenced the collection of waste, the improvement of health, the awareness of what one should be done with that organic waste, and that that waste right in the end is not exactly waste. It is waste that transforms and returns to the earth and becomes food again. I think that was essential for the community (Interviewee #9).*

#### Code 2: Diversity of knowledge

Considering only code 2, the subgroup “Collective learning” appears 62.4% of the time, thus being the most frequently identified response. Here, collective learning is also understood as any type of activity with different members of the community or external organizations collaborating in one activity that generated new knowledge, tacit knowledge, behaviours, or opportunities. This subcode is not only linked to the learning process of environmental issues, but it is evidence of a group with strong social bonds, a network that creates a learning environment for the development of knowledge in different areas (such as community identity or composting).

Regarding the subcode “Socio-ecological system thinking” (22.8%), the systemic view of the BRP is exemplified as:

…*the systemic vision of health has been very important. We are no longer just in sanitation technology or agriculture. The discussion about agrotoxics when it was only in the field of agricultural sciences was a very unfair dispute. With the coming of the public health vision this opened a very important perspective to make this dispute and overview and interconnections (Interviewee #7).*

*Because BRP happens with the separation of the waste at the source where the waste is being produced, which is the houses, the restaurants where the organic waste is produced is where there has to be the first separation of the waste, so this requires a change of habits that is only through education that occurs. BRP also has artistic developments, you know? They did a rap on BRP, at the time I was working there I also had the construction of vertical gardens, there were some artistic initiatives that they also have an educational role, you know? This story of mine occurred in 2012-13, because that's when I worked on the project. Now I don't know how the project is progressing* (Interviewee #8).

In these segments, it is possible to identify the environment as an extension of health, education, and arts, where organic waste and composting is a key element for closing the learning cycles.

#### Code 3:

Considering code 3, “Critical reflection”, the interviewees demonstrated a remarkable critical reflection on different topics, especially regarding societal structures that act upon the community, such as: income levels, gender, racism, classism, and access to health care, security, and education. For the interviewees (categories past and current BRP members), the BRP is a project that brings knowledge and space for a wider debate about community problems and possible transformations. As interviewee #11 states, '*We understand that only through conscious and transformative political action can the much-needed historical acceleration be promoted, thus aiming at the attainment and guarantee of the fundamental rights provided for in Article 5 of the Federal Constitution of 88.'* Therefore, the learning processes also follow a motivation of obtaining fundamental rights like health and food.

The overall reflections were holistic and the Paulo Freire method was frequently mentioned by the educators and staff members. The interviews presented knowledge of popular education processes. Moreover, data showed pieces of evidence that the project used different areas of knowledge for disseminating urban agriculture and debating environmental issues by applied theatre, politics, and gender studies.

*(BRP) is an action, first of all, because it is systemic, it will not allow us to talk about separating the organic without talking about the whole context. Second, because it is a transformation from the pedagogical educational process that happens in practice. So, people see the results in practice. And the residue that leaves the house that goes to compost is transformed into earth and this earth goes to the vegetable garden that produces a seasoning, a tea, a plant. Thus it is very concrete, it has a lot of concreteness (Interviewee #7)*

In code 3 for the subcode “Critically assessing the education in the community”, it identifies the recurrence of the statement mentioning the lack of attention to schools (public education in general) received from the local government. The criticism of the schools and education possibilities were frequently mentioned with a political issue linked to it, such as low investment/ budget for the schools, lack of sport areas and courses; lack of sexual education and gender (especially for girls); police attacks on young boys; drug trafficking; and others. Still in code 3, the subcode “Teaching ideas for other communities” was mentioned 10% of the time. Several segments mentioned their organizational and identity processes as a waste management model that could be used for other similar communities.

*So, I believe that communities have to articulate, they have to be understanding that they are in the same boat and looking for ways to evolve together, and so how to nourish these communities, these areas is to understand that to stop killing ourselves and several other things…* (*Interviewee #12).*

Finally, regarding the results of the complementary methodological phases 3 and 4, analysis of social media communication and participant observation, it shows that BRP is creating a community of organizations and professionals that contribute to enriching the field of community organic waste management and urban agriculture in Florianopolis. Moreover, the narratives associated with communication with external actors are associated with community empowerment. Not only is the BRP Instagram a channel to disseminate knowledge, but it creates a narrative of collective action being attributed to it. For example, this can be observed in the case of the post on 31 July 2019, when they posted a whiteboard, the result of an activity they created together with different visual and textual content about the BRP, it states: *"composting in order to transform our reality and territory!" and also "sensitize, articulate, deliver"*. There are eight direct posts showing learning activities and four of children’s workshops, reinforcing the data that evidence-based learning processes take place in the community. Furthermore, the 17 posts of people working together on-site show a level of organization and engagement, backed by the 14 posts showing community engagement in organic residues management.

## Discussion

Despite decades of sustainability research, a gap remains between theory and practice (Bonatti, 2018; Tseng et al., 2020). The results present advancement in the matter of applying sustainable and inclusive actions. One main result that points to the successful implementation of action and knowledge are the development of an identity as a motor to implement community transformations. Therefore, regarding how social learning was practically developed and based on which knowledge system (RQ1), data shows that BRP demonstrates several elements of social learning associated with the generation of knowledge that goes beyond simply understanding waste management. The knowledge generated is associated with several aspects of sustainability issues, such as food production, and is supported by critical reflection about community conditions/reality. These results also support our hypothesis that the social learning process was essential to organize knowledge systems related to environmental issues at the community level and beyond.

Another crucial aspect is that BRP is a platform for different kinds of knowledge: (a) it offers a learning process about waste management to the public and members (b) it creates and maintains a platform for knowledge creation, exchange, and critical reflection in the topics of human rights, environmental justice, and community identity.

The knowledge system is directly related to significant issues for the community. While the social learning process was initiated by analysing a concrete community problem, it was further established and improved based on the development of community identity. Theoretically, the social learning system developed by BRP can also be associated with coding and decoding (Bonatti, 2018; Freire, 2018). According to Freire (2018), knowledge representation (codification) and interpretation (decoding) are not mere instructional strategies rather these are the core of education and learning through praxis, to change reality according to the interests of its participants, who are active throughout the whole process. This type of structured educational methodology empowers the community to adopt the measures necessary for changing their reality through a deliberative process. Social learning is intrinsically participative and active, therefore capable of changing cultural practice towards sustainability (Bonatti et al., 2021).

In the context of criticizing their realities and fostering transformation, the community relate their identity to resistance to the conventional systems (food, political, educational) that have marginalized them. Participants of BRP adopted urban agroecology also as a political choice, one that gives space to resist the unequal structures. As the majority of the participant are women, and agroecology is a system that focuses on multiple elements all the food production system, it contributes to providing visibility to work developed by women, which is fundamental for the system’s sustainability and for the family’s reproduction (Carvalho, & Bógus, 2020). Therefore, in the context of Chico Mendes community, urban agroecology can be linked to feminism, going beyond the instrumental view of food production, as it brings practices and reflections that contribute to women’s struggles for rights, and emancipation from cultural and historical oppression (Altieri, 2012; Carvalho, & Bógus, 2020).

Regarding the analysis of to what extent social learning processes occur in the BRP (RQ2), we identify that the knowledge generated in the project has reached the three loops of learning. For instance, the critical reflection (second and third loop) (Medema et al., 2014) by community members and external actors helps to ensure robust knowledge about how and why to do urban agriculture but also integrates a necessary plurality of views, responsibility-sharing, and trust enhancement. The transformation (part) of community conditions is promoted by a dialogic interaction and creating space for ontological pluralism. For Souza et al. (2019), these are key principles of collective transformative learning. Education is a fundamental part of the process of transforming society towards sustainability. Therefore, there is a social demand for flexible, accessible, and reality-connected education (Abad-Segura & González-Zamar, 2021). Social learning fits within this social demand for education.

Results from the categories “past or current members of the BRP'” show a high percentage of learning schemes, more specifically at a community level and less among external actors. According to community members, a lack of trust has a high impact on developing collective action that includes the local government. The local government has a pivotal role in supporting the provision of waste management in the city. In this sense, public policies that promote waste management, alongside these community outreach strategies, could contribute to overcoming stigma and marginalization (Tremblay & Gutberlet, 2012) in other neglected communities.

**Fig. 4:**
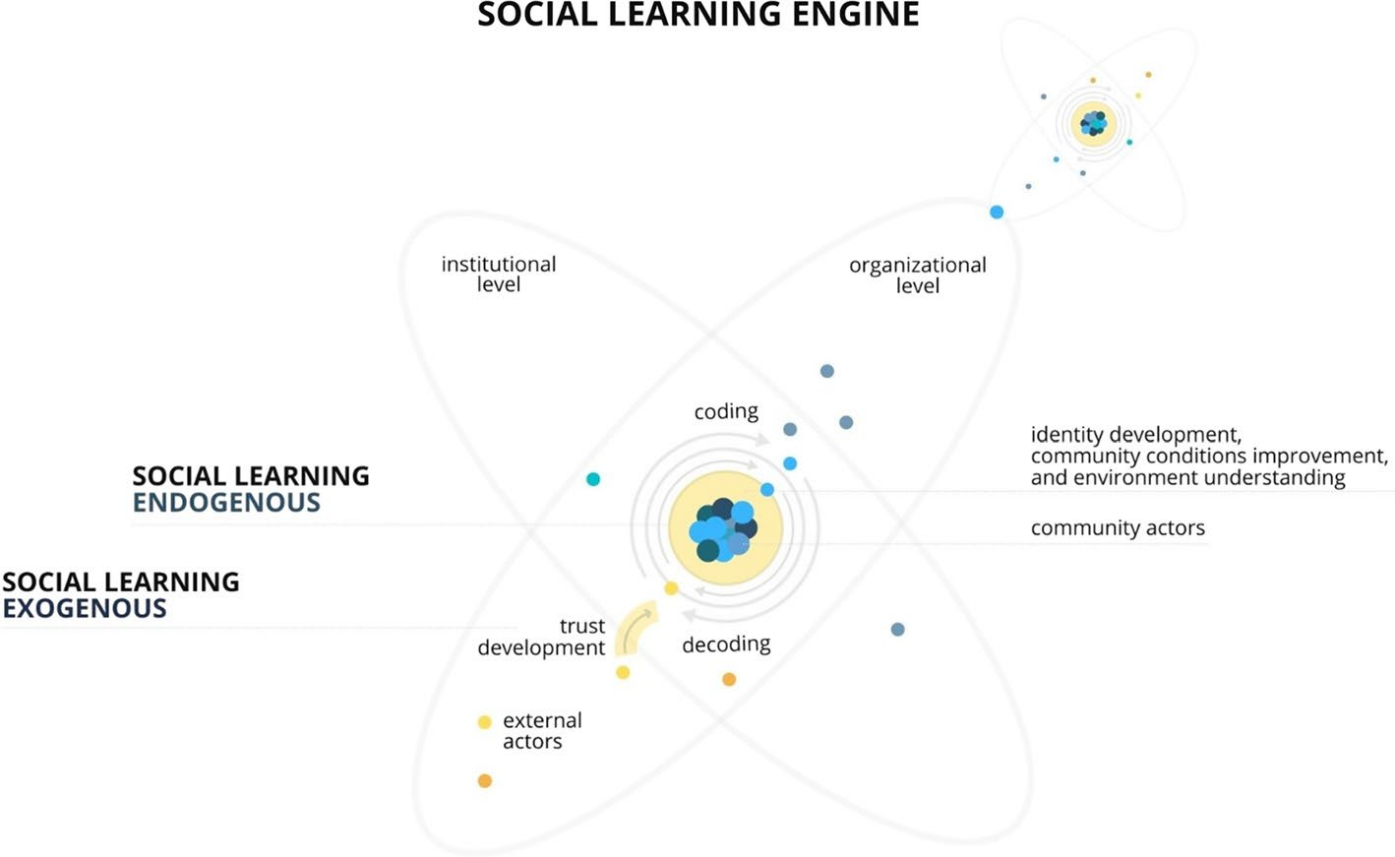
Engine of SL in the BRP

These different levels/orders of social learning identified are understood in this study as endogenous and exogenous. As proposed in Fig. 4, the centre of social learning at the community level is based on mechanisms related to the coding and decoding process. In this process, critical reflection focuses on the significant community issues as a driver of learning. Furthermore, the centre of this learning process is connected to identity construction. When this process is expanded by integrating the knowledge of external actors, exogenous social learning occurs.

The analysis of the BRP shows that the first driver for environmental change was principally a social issue, community health. In this sense, in neglected communities, responses to environmental problems should focus on community issues, integrating issues that are significant to the populations. These responses need to focus on the root causes, the underlying drivers of environmental changes, rather than only the pressures or symptoms (Joyeeta, et al., 2020). Neglected communities, understood as adaptive systems (Neely, 2015), could benefit from this systematization of social learning processes. This is because identifying key factors for improving adaptive capacity, identity sense, and waste management through learning is essential for better coping with the multiple currents and future threats that these communities face (Johannessen et al., 2019).

Regarding further research, as empirical data of this study showed, there are different kinds of social learning to be better investigated. In this sense, and following the studies of Ison et al. (2013) about metaphors of social learning, other metaphors and/or archetypes of social learning in natural resources management should continue to be investigated.

## Conclusions

Research on mechanisms to foster decentralized organic residue management is crucial to enhance the knowledge about how to solve one of the current biggest societal challenges: waste management. Therefore, research on this topic is urgent. Through empirical research, this article reveals interconnections between social learning theory and aspects of the coding and decoding concepts. This can be considered an advance for social learning theoretical rooting. Social learning is conceptually abstract, and its feasibility needs to be strengthened through a systematization of “real world” experiences like BRP.

As stated by several authors (Medema et al., 2014; Reed et al., 2010; Tosey et al., 2012), conceptualizations of triple-loop learning are diverse, often have little theoretical rooting, are sometimes driven by normative considerations, and lack support from empirical research. Therefore in terms of theoretical implications, by exemplifying triple-loop learning in empirical research, this article also contributed to advancing social learning theory to deal with complex sustainability problems. In terms of practical implications, the finding of this study can support policymakers to promote learning through decentralized organic waste management systems more connected to neglected community realities.

In the Chico Mendes community, the complex situation, which involves multiple problems, is improved by a knowledge system based on community identity development. The study of BRP social learning processes shows that significant community issues should be considered as triggers for collective action and transformative processes regarding natural resource management in neglected communities. When fundamental problems for the participants are fully considered, the process of social learning occurs.

In the BRP case, the three key components of the knowledge system enhanced through an underlying process of social learning are identity development, improved community conditions, and environment understanding. Although coordinated by a few people, BRP has managed a massive impact in the community in which it has emerged. Having managed tons of organic waste and contributed to the production of food for participating families, benefitting hundreds of people, the initiative relies on a learning system that goes beyond understanding waste management. The participants show that by using waste management as a starting point, the BRP facilitated a comprehensive platform of transformative collective learning about their community’s needs, rights, and sustainability.
